# Targeting Age-Related Impaired Bone Healing: ZnO Nanoparticle-Infused Composite Fibers Modulate Excessive NETosis and Prolonged Inflammation in Aging

**DOI:** 10.3390/ijms252312851

**Published:** 2024-11-29

**Authors:** Do-Yeun Kim, Jeong-Hyun Ryu, Jae-Hyung Kim, Eun-Hye Lee, Jeong-Hwa Baek, Kyung Mi Woo

**Affiliations:** 1 Department of Molecular Genetics, School of Dentistry and Dental Research Institute, Seoul National University, Seoul 08826, Republic of Korea; 2 Department of Pharmacology & Dental Therapeutics, School of Dentistry, Seoul National University, Seoul 08826, Republic of Korea

**Keywords:** aging, bone healing, transcriptomic analysis, NETosis, ZnO-infused polymer fibers

## Abstract

Bone defects present significant challenges in clinical contexts, particularly among the elderly, and are often linked to altered innate immune responses; however, underlying mechanisms remain to be understood. This study investigates immune changes in early bone healing in aged mice, emphasizing the effects of zinc in modulating inflammatory processes. By exploring the role of zinc and NETosis in this process, we seek to develop novel therapeutic strategies that could improve bone repair in aging populations. Critical-sized calvarial bone defects were induced in young (8-week-old) and aged (18-month-old) mice, with RNA sequencing analysis. Zinc oxide nanoparticle-infused polycaprolactone (ZnPCL) scaffolds were then fabricated using electrospinning, and their effects on intracellular zinc levels, NETosis, M2 polarization, and bone formation were assessed through in vitro and in vivo experiments. In aged mice, bone healing was delayed, inflammation was prolonged, and NETosis was excessive. RNA sequencing identified alterations in zinc ion transport genes, alongside excessive NETosis. Aged mouse neutrophils exhibited low intracellular zinc levels. ZnPCL fibers effectively reduced NETosis and inflammation, promoted M2 macrophage polarization, and enhanced new bone formation, thereby improving bone healing in aged mice. This study demonstrates that ZnO nanoparticle-infused biomaterials, ZnPCL, effectively deliver zinc to neutrophils, reduce NETosis, promote M2 polarization, and enhance bone healing in aged mice.

## 1. Introduction

The global population aged 65 and over is projected to nearly double, reaching approximately 16–17% of the total population by 2050 [1]. This demographic shift underscores the expanding elderly population’s impact on various societal sectors, including healthcare. Moreover, it has long been recognized that the physiological systems in elderly individuals undergo significant changes, resulting in altered pharmacokinetic and pharmacodynamic responses to drugs compared to younger adults [2]. Given the projected growth in age-related conditions and the substantial differences between aged and young populations that could impact responses to treatments, there is a pressing need for preemptive strategies to address the healthcare demands of the elderly.

Among the various health issues associated with aging, bone health stands out as particularly crucial. In this context, elderly individuals face an increased risk of fractures due to a decrease in bone mineral density and a rise in osteoporosis [3,4,5]. These conditions not only lead to a higher incidence of bone defects but also result in prolonged recovery periods. Consequently, there is a vital need for research focused on enhancing bone healing in the elderly, considering the significant challenges such as increased mortality, reduced mobility, and heightened complications that accompany delayed or incomplete bone recovery. Impaired bone regeneration in the elderly is attributed to several factors, including enhanced marrow adiposity, stem cell aging, altered mesenchymal stem cell differentiation, and shifts in inflammatory and innate immune responses. Specifically, aging-related changes in the immune system significantly affect defect healing [5,6,7]. While bone cell metabolism genes remain relatively stable with age [8], immune system alterations, particularly immune cells in the bone marrow, contribute to compromised bone defect healing [5,9].

Transcriptomic analysis has become a powerful tool for advancing our understanding of complex biological processes and discovering new therapeutic targets. This study analyzed the transcriptome of early bone healing in aged mice, with a focus on identifying immune-related targets. Based on transcriptomic analysis and histological observation, we noticed and explored the role of zinc and NETosis (neutrophil extracellular trap formation) during early bone wound healing. NETosis is a form of programmed cell death, specifically occurring in neutrophils [10]. During NETosis, neutrophils release web-like structures called neutrophil extracellular traps, which are composed of decondensed chromatin and granule proteins like neutrophil elastase and myeloperoxidase. While NETosis is a critical defense mechanism against infections, its dysregulation can contribute to a variety of inflammatory conditions. Uncontrolled NETosis can lead to persistent inflammation and also contribute to tissue damage [11,12]. Bio-inorganic zinc is integral to bone health, immune function, and wound healing as an essential trace element [13,14]. Zinc deficiency, more common in older adults, is linked to slower wound healing and immune system dysfunction post-injury [15,16]. Addressing zinc deficiency is crucial for supporting overall health and bone healing in the elderly [15,16,17]. Zinc regulates the activity of protein arginine deiminase (PAD) 4, a critical molecule for NET formation [18]. Previous studies have shown that zinc can aid in bone healing [19,20], but its effects have not been studied in the context of aging, which brings significant changes, as seen in aged animals.

This study introduces a zinc oxide (ZnO)-infused polymer composite fibrous scaffold, developed under the hypothesis that zinc-enriched biomaterials can enhance bone healing in the elderly. Our research aims to investigate the relationship between delayed bone defect healing and factors like excessive NETosis (formation of neutrophil extracellular trap) and prolonged inflammation. Through in vitro and in vivo experiments, we explored the ZnO-infused composite fibrous scaffold’s effectiveness, offering insights into potential interventions for improved bone health in the aging population.

## 2. Results

### 2.1. Impaired Bone Healing in Aged Mice

**Figure 1:**
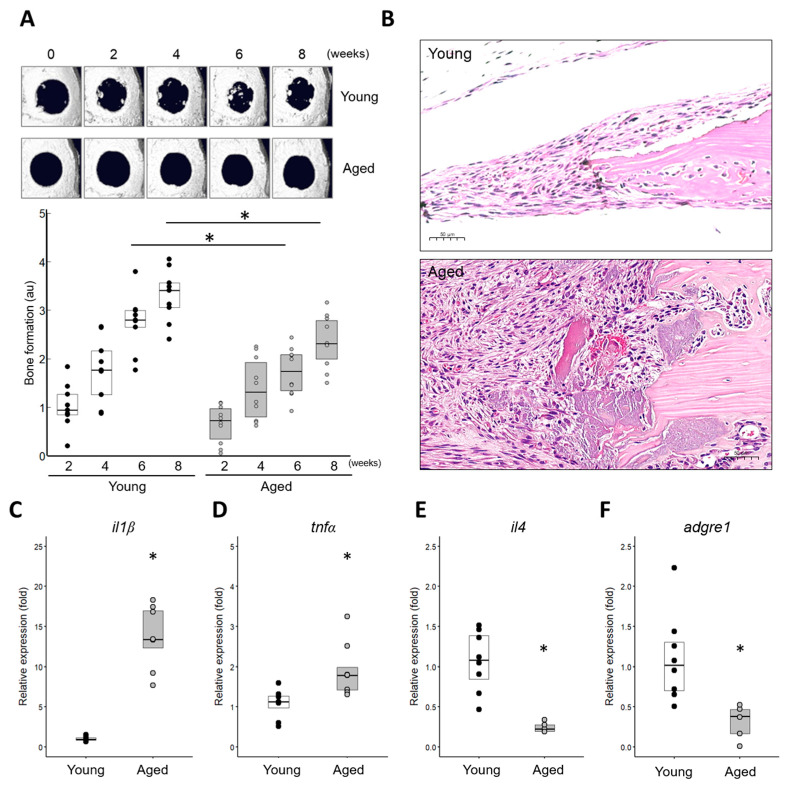
Comparative analysis of bone defect healing in aged mice versus in young mice. (**A**) Reconstructed microCT images showing the time/course of bone healing following calvarial bone defect surgery with a critical-sized 6 mm defect. Newly formed bone volumes were quantified (*n* = 9 individual samples). * Significant difference in young and aged (*p* < 0.05). (**B**) Histology of bone defect wounds at 7 days post-operation. Microphotographs were captured at an original magnification of 20× (scale bar: 50 μm). (**C**–**F**) Expression of pro-inflammatory cytokines (*il-1β* and *tnf-α*), an anti-inflammatory cytokine (*il-4*), and a monocyte/macrophage marker (*adgre1*, also known as *F4/80*). Total RNA was extracted from tissues at the bone wound sites 7 days post-operation (young: *n* = 8, aged: *n* = 5). * Significantly different from young (*p* < 0.05).

A critical-sized defect (6 mm in diameter) was surgically created in the calvarial bones of mice, and the healing process was monitored for up to 8 weeks. In live microCT imaging, aged mice (18 months or older) exhibited impaired bone healing, as evidenced by significantly reduced bone volumes compared to young mice (Figure 1A). Histological features revealed a significant infiltration of inflammatory cells in the wound areas of aged mice, with polymorphonuclear cells (PMNs, mainly neutrophils) persisting in older mice while decreasing in younger mice to day 7 (Figure 1B). To investigate the characteristics related to inflammation in the wound areas of the aged, levels of pro-inflammatory cytokines (*il-1β* and *tnf-α*) (Figure 1C,D) were checked and confirmed to be elevated. In contrast, an anti-inflammatory cytokine (*il-4*) and a monocyte/macrophage marker (*adgre1*) were expressed at much lower levels (Figure 1E,F). These results indicate that aged mice exhibit impaired (delayed) bone healing and prolonged inflammation, consistent with the findings in previous reports [6].

### 2.2. Exploring the Potential Link Between Zinc, NETosis, and Impaired Bone Healing in Aged Mice

**Figure 2:**
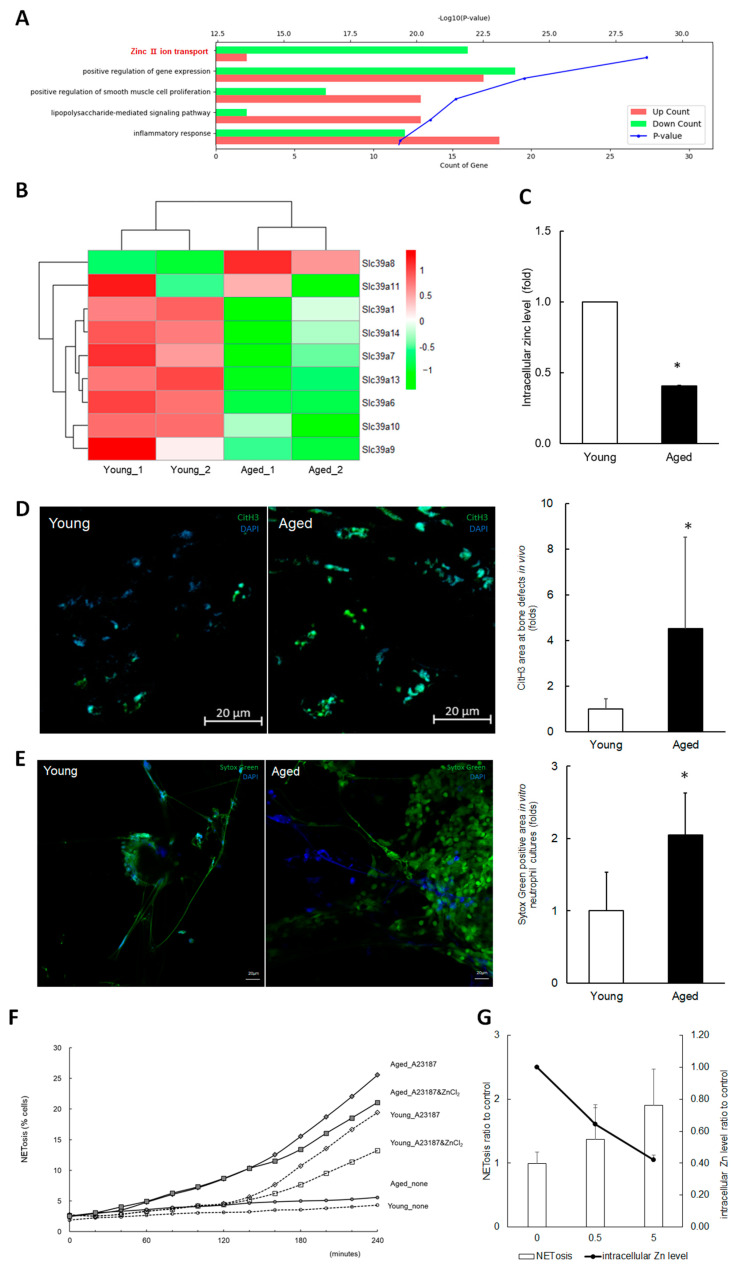
Exploring the association between zinc levels, NETosis, and impaired bone defect healing in aged mice. (**A**) Gene ontology analysis from RNA sequencing. RNA samples were extracted from tissues of calvarial bone defects at day 1 post-operation in young and aged mice. Green bars represent genes with decreased expression in aged mice, while red bars represent genes with increased expression. The blue dotted line represents the *p*-value. (**B**) A heatmap of gene expression Z-scores for zinc transport genes based on RNA sequencing data from day 1. (**C**) Intracellular zinc levels in neutrophils isolated from young and aged mice were measured. FACS analysis was performed on Fluozin3-stained cells, and the results from three independent assays are presented as fold change values. (**D**) Immunofluorescent images of citrullinated histone H3 (citH3), a hallmark of NETosis. Microphotographs of the bone wound sites 7 days post-operation were captured at four random sites from three slides (original magnification: 40×, scale bar: 20 μm). The citH3 area was quantified and presented as normalized values with DAPI. * Significantly different from young (*p* < 0.05). (**E**) Induction of NETosis in the neutrophils isolated from aged and young mice. The neutrophils treated with A23187 were stained with SytoxGreen and DAPI. Immunofluorescent microphotographs were captured at an original magnification of 20× (scale bar: 20 μm). SytoxGreen positive area was measured and presented as normalized values with DAPI. * Significantly different from young (*p* < 0.05). (**F**) A time/course profile of NETosis induction. Isolated neutrophils were incubated with ZnCl_2_, induced to undergo NETosis with A23187, stained with SytoxGreen, and monitored for up to 240 min using Image Exfluore 5.21. (**G**) Effect of TPEN on NETosis induction. Neutrophils were incubated with TPEN and A23187 for 4 h. * Significantly different from control non-treated group (*p* < 0.05).

RNA-seq was conducted in the young and aged mice during the initial stages of bone healing (up to 7 days) in order to explore the effects of aging in the context of bone healing. The transcriptomic analyses revealed significant changes in GO terms, including zinc ion transport and inflammatory response on days 1, 4, and 7 (Figure 2A). The heatmaps of gene expression Z-scores for zinc transport genes also showed significant differences between young and aged samples (Figure 2B). The zinc ion transport, especially influx, genes were downregulated in the aged mice group, which was also validated by RT-qPCR. In primary neutrophil cultures isolated from the bone marrow of mice, it was confirmed that levels of intracellular zinc ions (Figure 2C) were significantly lower in aged mice compared to younger ones.

The literature indicates that excessive NETosis leads to prolonged inflammation, adversely affecting wound healing processes [21]. In this study, NET levels were examined in aged mice during the early stages of bone defect healing and compared to their younger counterparts. Citrullinated Histone H3 (citH3) staining indicated approximately four times higher NET formation in the bone wounds of aged mice than in younger ones (Figure 2D). In cultures of primary neutrophils isolated from bone marrow, SytoxGreen staining for extruded DNA highlighted roughly double the NET formation in neutrophils from aged mice upon induction of NETosis with A23187, compared to those from younger mice (Figure 2E). These in vivo and in vitro results indicate that NETosis is increased in bone defect wounds of aged mice, suggesting an association between excessive NETosis and impaired bone healing in these mice.

There have been previous studies that have suggested zinc’s role in modulating the immune system’s involvement in excessive inflammation [14] and impaired bone healing [7]. In addition, it has been also reported that zinc interferes with the activity of PAD4 and NET formation [18,22,23]. Thus, the effect of zinc on NET induction in primary neutrophils was examined. Adjusting zinc levels with ZnCl_2_ or a zinc chelator TPEN showed that TPEN heightened NET formation, whereas ZnCl_2_ reduced NETosis (Figure 2F,G), as verified by SytoxGreen staining and Image Exfluore analysis. These results suggest that zinc-releasing biomaterials could reduce excessive NETosis and improve bone healing in aged individuals.

### 2.3. Characterization of ZnO-Infused Poly(ε-Caprolactone) Composite Fibers

**Figure 3:**
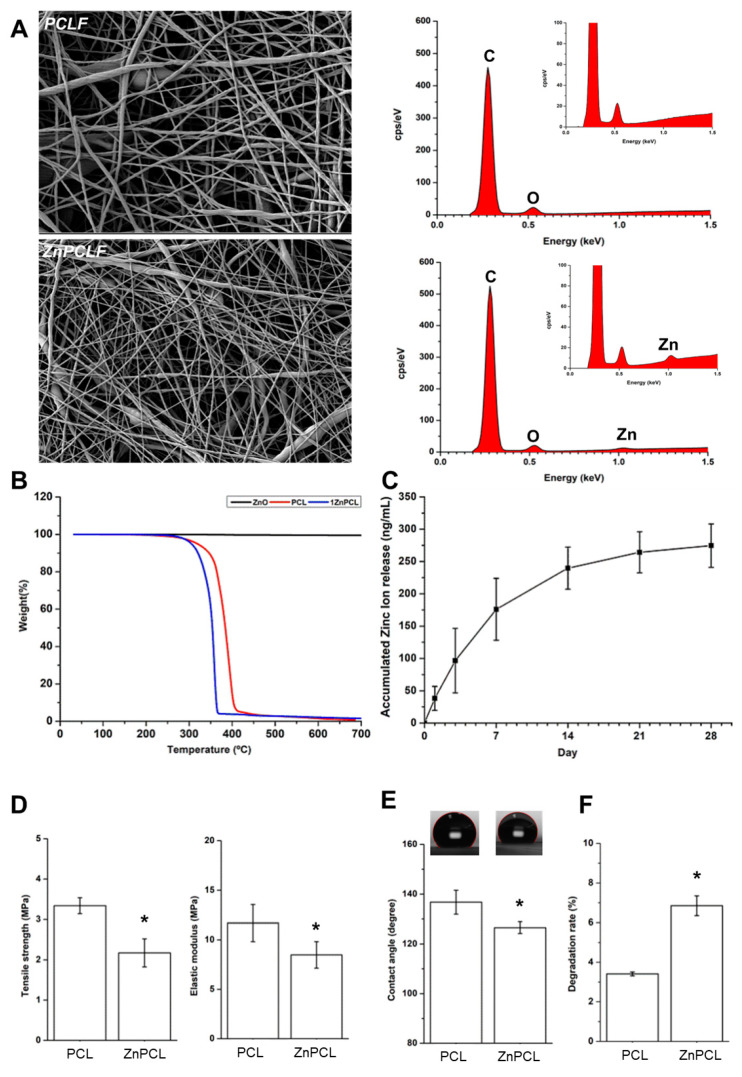
Physicochemical characterization of ZnPCL composite nanofibers in comparison with PCL. (**A**) SEM images (left) and EDS analysis (right). (**B**) Thermogravimetric analysis of ZnO, PCL, and ZnPCL. (**C**) In vitro release of zinc ion from ZnPCL composite scaffolds. The samples were immersed in simulated body fluid (SBF), and the amounts of zinc ions released to SBF were measured by inductively coupled plasma mass spectrometer. (**D**) Tensile stress and elastic modulus of PCL and ZnPCL. (**E**) Water contact angle of PCL and ZnPCL. (**F**) In vitro biodegradation of PCL and ZnPCL. The samples were immersed in SBF for 14 days at 37 °C, and molecular weights were determined by gel permeation chromatography. Data presented as averages and standard deviations (*n* = 6). * Significantly different from PCL (*p* < 0.05).

Based on our results, ZnPCL was developed. The zinc oxide nanoparticles used had an average zeta particle size of 40.61 ± 16.08 nm. The physicochemical properties of the electrospun ZnPCL nanofibers were analyzed alongside those of PCL nanofibers (Figure 3). Their surface structures were examined using SEM (Scanning Electron Microscope), and the PCL fibers had an average diameter of 197 ± 101 nm, while the ZnPCL fibers were measured slightly larger at 211 ± 109 nm. The incorporation of zinc within the ZnPCL nanofibers was verified using EDS (Energy-dispersive X-ray spectroscopy) and thermogravimetric analysis (Figure 3A,B). When submerged in SBF (simulated body fluid), the ZnPCL scaffolds began releasing zinc ions, demonstrating a steady release over 28 days, with a remarkable initial release of about 36% within the first three days (Figure 3C). This release pattern indicates the potential of ZnPCL to make an immediate beneficial impact on micro-environments at the onset of healing. The tensile strength and elastic modulus of the ZnPCL nanofibers were found to be lower than those of pure PCL nanofibers (Figure 3D). ZnPCL exhibited reduced hydrophobicity compared to PCL, as determined by water contact angle measurements (Figure 3E). Biodegradation tests in vitro further showed that ZnPCL degrades more swiftly than PCL (Figure 3F). Therefore, ZnPCL presents a promising avenue for positively influencing the early wound micro-environments by delivering zinc.

### 2.4. Effect of ZnPCL on NETosis in Neutrophils Isolated from Aged Mice

**Figure 4:**
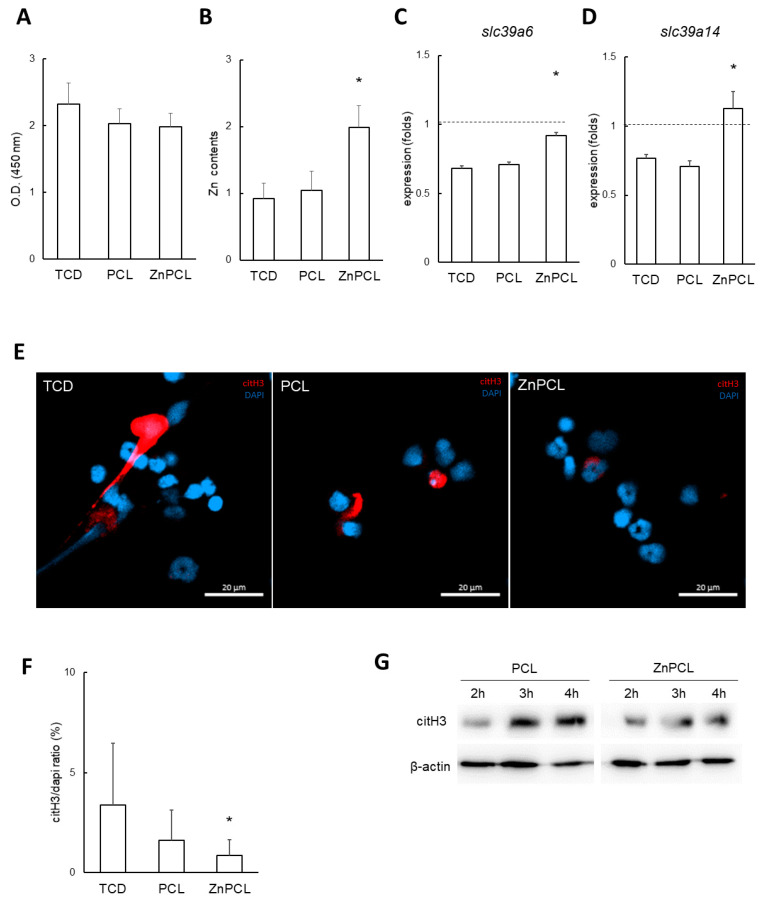
Effect of ZnPCL on NETosis in neutrophils isolated from aged mice. (**A**) Cell viability. An osteoblast cell line (MC3T3-E1) was cultured on tissue culture dishes (TCDs), PCL, and ZnPCL for 3 days. A WST-1 assay was performed to assess cell viability. (**B**) Intracellular zinc levels in neutrophils from aged mice. Neutrophils were incubated on TCD, PCL, and ZnPCL. Intracellular zinc levels were analyzed using a zinc assay kit (*n* = 5). * Significantly different from TCD (*p* < 0.05). (**C**,**D**) Expression of zinc transporters, *Slc39A6* and *Slc39A14*, in neutrophils isolated from aged mice. Horizontal dashed lines represent the levels of neutrophils from young controls (TCD). Data are presented as averages and standard deviations. * Significantly different from TCD (*n* = 3, *p* < 0.05). (**E**) Immunofluorescent images of citH3 in neutrophils isolated from aged mice. Neutrophils were induced to undergo NETosis with A23187 and stained for citH3. Microphotographs were captured at an original magnification of 40× (scale bar: 20 μm). (**F**) The citH3 area was quantified and presented as normalized values relative to DAPI. * Significantly different from TCD (*p* < 0.05). (**G**) Western blot analysis for citH3 in isolated neutrophils. β-actin served as the loading control.

In preliminary cell viability tests using the MC3T3-E1 osteoblast cell line, ZnPCL did not show a significant difference in cell viability compared to tissue culture dishes (TCDs) and PCL (Figure 4A). To evaluate the effects of ZnPCL on NETosis, primary neutrophils isolated from aged mice were cultured with TCD, PCL, and ZnPCL. The decreased intracellular zinc levels observed in aged mice were increased by approximately 216% by ZnPCL, restoring them to levels comparable to those in young mice (Figure 4B). Additionally, the expression levels of the zinc ion influx transport genes, *slc39a6* and *slc39a14*, which were downregulated in aged mice, were restored with ZnPCL treatment (Figure 4C,D). These results indicate that ZnPCL effectively adjusted intracellular zinc levels in aged mice. These results indicate that ZnPCL increased intracellular zinc levels in aged mice neutrophils compared to untreated controls.

The potential of ZnPCL to reduce NETosis in primary neutrophils from aged mice was investigated. Neutrophils isolated from aged mice were cultured and NETosis was induced using A23187. A significant reduction in induced NETosis was observed in ZnPCL-treated neutrophils, with levels reduced to approximately 25% compared to the control group (Figure 4E,F). Additionally, Western blot analysis of citH3 levels in neutrophils from aged mice revealed a time-dependent increase in citH3. In contrast, ZnPCL-treated neutrophils exhibited significantly lower levels of citH3 (Figure 4G). Collectively, these results indicate that ZnPCL effectively delivered zinc to neutrophils and reduced the induction of NETosis in neutrophils isolated from aged mice, suggesting that ZnPCL has the potential to mitigate the excessive NETosis observed in delayed bone healing of aged mice.

### 2.5. Effect of ZnPCL on NETosis, Macrophage Recruitment, and M2 Polarization in an In Vivo Bone Defect Model of Aged Mice

**Figure 5:**
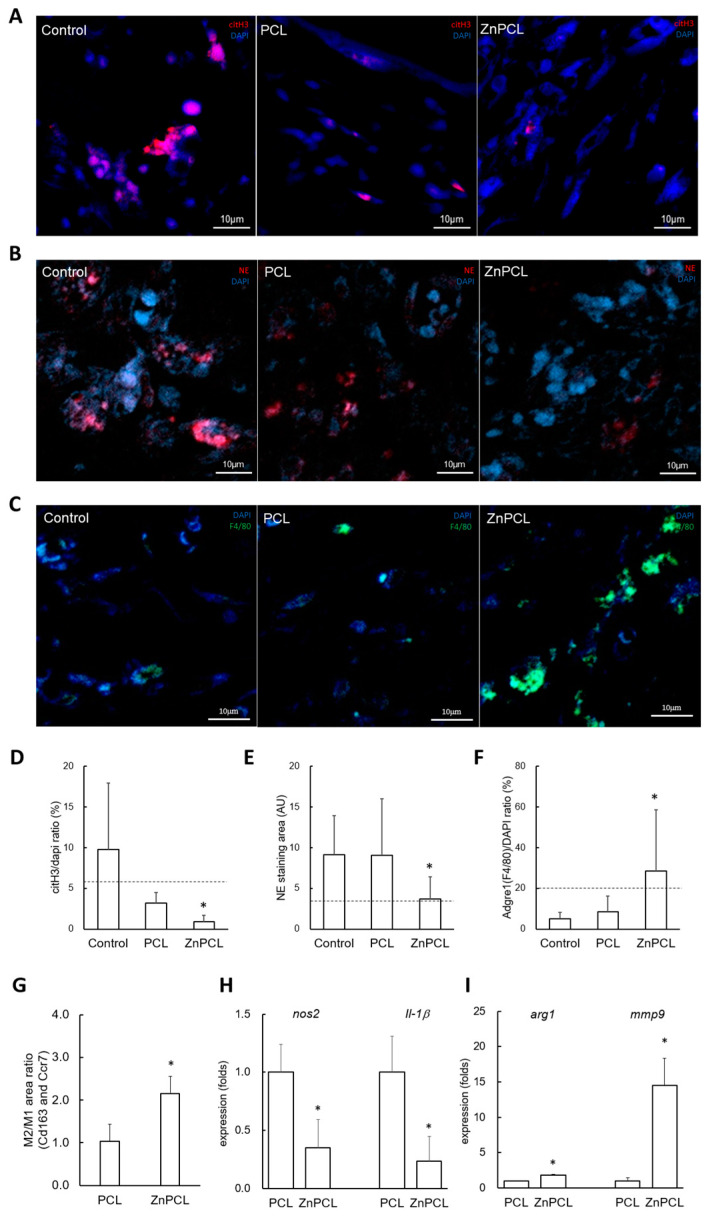
Effect of ZnPCL on NETosis, macrophage recruitment, and M2 polarization in an in vivo bone defect model of aged mice. The PCL and ZnPCL scaffolds were implanted into calvarial bone defects of 3 mm in diameter and analyzed 7 days after the operation. (**A**,**D**) NETosis. Immunofluorescent images for citH3 were captured at four random sites from three slides (original magnification: 40×, scale bar: 10 μm). The area of citH3 was quantified and presented as normalized values relative to the DAPI-stained area. A horizontal dashed line indicates the level of citH3 in the young control group. * Significantly different from the sham control group (*p* < 0.05). (**B**,**E**) Persistent neutrophil presence. Immunofluorescent images for neutrophil elastase (NE) were captured under the same conditions as in NETosis (original magnification: 40×, scale bar: 10 μm). The area of NE was quantified and presented as normalized values relative to the DAPI-stained area. A horizontal dashed line indicates the level of NE in the young control group. * Significantly different from the sham control group (*p* < 0.05). (**C**,**F**) Macrophage recruitment. Immunofluorescent images for F4/80 (Adgre1) were captured under the same conditions as in NETosis (original magnification: 40×, scale bar: 10 μm). The area of F4/80 was quantified and presented as normalized values relative to the DAPI-stained area. A horizontal dashed line indicates the level of F4/80 in the young control group. * Significantly different from the sham control group (*p* < 0.05). (**G**) Expression of M1 and M2 phenotype markers. The immuno-stained areas of Ccr7 and Cd163 were quantified and presented as normalized values relative to the DAPI-stained area. The ratio of Cd163 to Ccr7 was calculated from each double-staining image. * Significantly different from the sham control group (*p* < 0.05). (**H**,**I**) M1 and M2 phenotype markers. Total RNA was extracted from tissues at the bone wound sites 7 days post-operation and analyzed by RT-qPCR. Data are presented as averages and standard deviations (*n* = 3). * Significantly different from PCL (*p* < 0.05).

In a calvarial bone defect model with a 3 mm defect size, citH3 staining was performed for immunofluorescence imaging on day 7 post-operation. The citH3-positive area normalized with DAPI was approximately 164% higher in the control group in aged mice compared to their young counterparts. Notably, the ZnPCL-treated group in aged mice showed a significant decrease in the citH3-positive area, reducing it to approximately 8.8% of the level observed in the control group (Figure 5A,D).

To assess whether the inflammation phase exceeded typical durations, we analyzed samples from calvarial defects on day 7 post-operation. Initially, neutrophils act as the primary responders at the onset of inflammation, transitioning to anti-inflammatory M2 macrophages by day 7, which leads to a decrease in neutrophil presence (6, 23, 24). Immunofluorescence analyses to detect neutrophil elastase for neutrophils and F4/80 for macrophages indicated that persistence of neutrophil elastase was shown in the control and PCL groups, significantly reduced in the ZnPCL group, where an increase in macrophages was noted (Figure 5B,C,E,F). These findings imply that the inflammatory phase is extended in aged mice; however, ZnPCL treatment may expedite the resolution of inflammation.

To further confirm the inflammatory state and its association with macrophage polarization, we checked levels of M1 (Ccr7) and M2 (Cd163) macrophage phenotypes through immunostaining. The ZnPCL group exhibited an increase in Cd163 staining, leading to a significantly higher ratio of M2 (Cd163) to M1 (Ccr7), reaching approximately 208% compared to the control group (Figure 5G and Figure S1). Additionally, gene expression levels of M1 and M2 macrophage phenotypes were analyzed. The M1 markers, *nos2* and *il-1β*, were expressed at a lesser extent in the ZnPCL group. Conversely, the M2 phenotypes (*arg1* and *mmp9*), which represent an anti-inflammatory and pro-healing stage, were more prevalent in the ZnPCL group (Figure 5H,I). Collectively, these results highlight a prolonged active inflammatory phase in control groups in the aged group, whereas the ZnPCL group demonstrated a shift towards an anti-inflammatory phase.

### 2.6. ZnPCL Expedited Bone Healing in Aged

**Figure 6:**
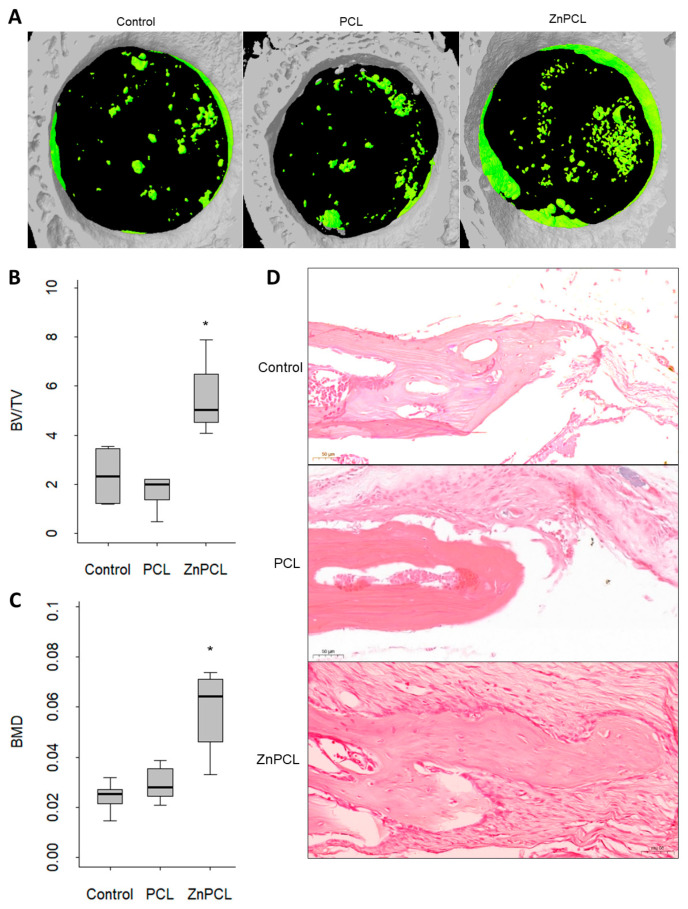
Effect of ZnPCL on bone healing in aged mice. (**A**) Reconstructed micro-CT images. Calvarial bone defects of 3 mm in diameter were created bilaterally in aged mice, which were sacrificed after 8 weeks. Newly formed bone is depicted as green. (**B**) % bone volume and (**C**) bone mineral density (BMD). Data presented as averages and standard deviations (*n* = 5~8). * Significantly different from control group in aged mice (*p* < 0.05). (**D**) Microscopic images of bone defect healing after 8 weeks of placing PCL and ZnPCL on defects. The images were captured at the original magnification ×20 (scale bar-50 μm).

In an experimental calvarial defect model, bone defects of 3 mm in diameter were created in aged mice and treated with either a non-treated sham control, PCL, or ZnPCL. At 8 weeks post-operation, micro-CT analysis assessing bone volume to total volume ratio and bone mineral density revealed that ZnPCL significantly increased both bone volume and bone mineral density in aged mice, bringing them to levels comparable to those in young mice (Figure 6A–C). Histological analysis confirmed these findings, consistent with the micro-CT results. The PCL group in aged mice did not show a noticeable difference compared to the non-treated control. However, the introduction of ZnPCL in aged mice significantly enhanced bone recovery (Figure 6D and Figure S2). These results demonstrate that ZnPCL facilitates impaired bone healing in aged mice in vivo.

## 3. Discussion

This study investigates the effect of aging on bone healing, focusing on the innate immune response, particularly the roles of zinc and NETosis in aged mice. We demonstrated that bone healing was delayed in aged mice, accompanied by increased inflammation, consistent with previous studies. Furthermore, our study introduces ZnPCL as a novel approach to enhance bone healing in aged mice. Using a calvarial defect mouse model, we showed that ZnPCL supports the recovery of impaired bone healing by reducing excessive NETosis and promoting M2 macrophage polarization. These findings indicate that ZnPCL not only mitigates excessive NETosis but also accelerates the bone healing process.

Aging complicates bone healing, and our research provides crucial insights into the underlying mechanisms, potentially laying the foundation for more effective treatments for the elderly. Aged mice with calvarial bone defects exhibited significantly less bone repair compared to young mice. Additionally, aged mice experienced excessive NETosis, prolonged inflammation, and delayed healing, all of which hinder the repair process. To understand the mechanisms underlying impaired bone healing, we employed RNA sequencing and transcriptomic analysis to identify candidates that could mitigate excessive NETosis and promote healing in aged mice. Analysis of bone wound beds revealed differences in gene expression profiles related to zinc transporters. Intracellular zinc levels in neutrophils from aged mice were much lower than those in younger mice, despite serum zinc levels being higher in aged subjects. Our findings confirmed that decreased zinc concentrations in neutrophils led to increased NETosis, while higher zinc levels reduced excessive NETosis.

NETosis, a process where neutrophils release extracellular traps composed of decondensed chromatin and cytotoxic proteins, has garnered significant attention in aging and various diseases [24]. While NETs play a defensive role, they can also cause tissue damage and impair wound healing. Aged mice produce more NETs, but these NETs are less effective [25]. Rejuvenating neutrophils has been shown to enhance bone healing in older subjects [24]. Chronic inflammation, a hallmark of aging [26], exacerbates conditions such as diabetes, where excessive NETosis impairs wound healing [21]. NETosis increases with age, likely due to elevated PAD4 activity, though the mechanisms remain incompletely understood [24]. NETosis formation involves inflammation-induced increases in intracellular calcium levels, which activate PAD4. PAD4 citrullinates histones, facilitating chromatin decondensation and NET formation [27]. Zinc ions can inhibit PAD4 activation by binding to it, thereby reducing NETosis [18]. Zinc supplementation has been shown to decrease PAD4 expression and NETosis while promoting anti-inflammatory cytokine production [28]. These studies suggest that zinc directly regulates PAD4 activity and NETosis formation.

Zinc is involved in regulating inflammation through various mechanisms, further supporting ZnPCL’s potential benefits. In a lung injury mouse model, zinc supplementation reduced neutrophil recruitment and NETosis formation [29]. Zinc deficiency affects other immune cells, including monocytes, macrophages, T cells [14], and intestinal epithelial cells, by increasing neutrophil migration through membrane damage and enhancing chemokine secretion [30]. ZnPCL is infused with ZnO, which has demonstrated anti-inflammatory, antioxidant, and anticancer properties [31,32,33]. The nanofiber inherently possesses immunomodulatory effects through its structural characteristics, surface design, and physical cues, independent of any ZnO-specific effects [34]. The oxidative stress theory of aging suggests that accumulated oxidative damage contributes to aging, positioning antioxidants as therapeutic strategies [35]. Zinc-modified hydroxyapatite reduces neutrophil-driven inflammation by downregulating IL-8 and CXCR-1 expression, minimizing ECM degradation [36]. Similarly, ZnO lowers cytokine levels and regulates neutrophil activity in viral infections [37]. However, excessive ZnO exposure may induce oxidative stress, particularly at high concentrations or with prolonged use. ZnPCL addresses this risk by providing localized and controlled zinc release, maximizing benefits while minimizing adverse effects. This design makes ZnPCL a promising material for mitigating inflammation and supporting tissue regeneration, particularly in aged environments.

The systemic effects of zinc release must be carefully managed. Zinc toxicity is typically associated with serum zinc concentrations exceeding 200 μg/dL, leading to gastrointestinal disturbances, immune dysfunction, and copper metabolism disruptions [19,38,39,40,41,42]. Long-term zinc alloy implants have been linked to severe inflammation, highlighting the need for surface modifications and optimized composites [20,42]. ZnPCL, designed for localized zinc delivery, minimizes systemic toxicity risks, as shown by its controlled release profile (Figure 3). However, further validation using appropriate models is necessary to ensure its safety for clinical applications. ZnPCL scaffolds hold significant potential for bone regeneration in elderly patients. PCL-based materials are already widely used in FDA-approved medical applications, such as tissue engineering scaffolds, dermal fillers, and biodegradable sutures [43]. For ZnPCL, additional studies are required to address critical factors such as toxicity, biodegradability, optimal dosing, and regulatory compliance. These analyses will be essential to translate ZnPCL scaffolds into safe and effective clinical treatments for this vulnerable population. In summary, in the calvarial defect model of aged mice, ZnPCL reduced inflammation and influenced macrophage polarization, accelerating bone healing. Our findings emphasize the importance of inflammation and NETosis in bone repair, particularly in the early phases of healing. Our investigation contributes to the development of targeted treatments for elderly individuals by advancing therapeutic strategies for bone regeneration, with a focus on addressing age-related inflammation and delayed healing processes.

## 4. Materials and Methods

### 4.1. Animal Study

All the procedures adhered to the guidelines outlined in the IACUC Guide for the Care and Use of Laboratory Animals and received approval from the Institutional Animal Care (SNU-230508-1-3). Male C57BL/6 mice, aged 2 months and 18–24 months, underwent the creation of bone defects in the parietal bones: either a single 6 mm circular defect for monitoring comparative bone healing (Figure 1) or bilateral 3 mm circular defects for evaluating responses to PCL, ZnPCL, or no treatment (Figure 5 and Figure 6). Bilateral injuries were either left untreated (sham control), placed with PCL scaffolds, or with ZnPCL scaffolds.

### 4.2. microCT

Eight weeks post-surgery, mouse skulls were fixed in paraformaldehyde and subjected to microCT imaging using Skyscan1272 (Bruker, Kontich, Belgium). After capturing the 3D data, a volume of interest was defined within a 3 mm diameter circle to measure total volume, bone volume, and bone mineral density based on the thickness of bone. The measurement of bone mineral density utilized the QRM-MicroCT-HA D32 Phantom.

### 4.3. H-E and Immunofluorescence Staining

At days 4 and 7, and week 8 post-surgery, the dissected sections of the skull were embedded in paraffin blocks for H and E staining, or immunofluorescence staining. The creation of paraffin blocks from the samples and the H and E staining were conducted. For immunohistostaining, the primary antibody Recombinant Anti-citH3 (Abcam, Cambridge, UK) was applied, and secondary antibodies Donkey Anti-Rabbit IgG (Alexa Fluor^®^ 568, Abcam) for citH3 were used. A Cy5 conjugated polyclonal antibody against neutrophil elastase, (Abcam) was used for NE staining. After applying the mounting medium with DAPI (ibidi, Gräfelfing, Germany), the samples were captured by a Confocal Laser Scanning Microscope (LSM 800, Carl Zeiss, Oberkochen, Germany).

### 4.4. Total RNA Sequencing

Calvarial defects were prepared, and the tissues from the defect area were dissected and immediately placed on ice 1, 4, and 7 days post-surgery. Total RNAs were extracted using QIAzol Lysis Reagent (QIAGEN, Valencia, CA, USA). The library was prepared using the Illumina TruSeq Stranded Total RNA Library Prep Gold Kit (Illumina, Inc., San Diego, CA, USA). From the total RNA sample, rRNA was depleted to detect both mRNA and lncRNA expression. Following rRNA depletion, samples were fragmented into small pieces, and first-strand cDNAs were copied using SuperScript II reverse transcriptase (Invitrogen, Carlsbad, CA, USA) and random primers. Second-strand cDNA was synthesized by adding DNA polymerase I, RNase H, and dUTP. Subsequently, the products were purified and enriched with PCR to create the final cDNA library. Indexed libraries were submitted to Illumina NovaSeq6000 (Illumina), and the paired-end (2 × 100 bp) sequencing was performed by Macrogen (Seoul, Republic of Korea). We performed Differential Expression Analysis (DEA) using DESeq2 (v1.32.0). For identifying differentially expressed genes (DEGs) within the “treat” group, each condition was compared against the “control”. In Figure 2, the top 5 Gene Ontology (GO) terms with the lowest *p*-values were selected, excluding those with a fold change of 9 or below. Over-Representation Analysis (ORA) was conducted using GO terms to identify biological pathways associated with DEGs. Annotation was performed using the org.Mm.eg.db (v3.17.0) and msigdbr (v7.5.1) databases. EnrichGO and gofilter functions from clusterProfiler (v4.8.1) were utilized for ORA.

### 4.5. RT-qPCR

Total RNA was extracted. The cDNA was synthesized using AccuPower^®^ RT PreMix (Bioneer, Daejeon, Republic of Korea), and RT-qPCR analysis was performed using TB Green Premix Ex Taq II (Takara, Kusatsu, Japan). The expression levels of target gene mRNAs were normalized to glyceraldehyde-3-phosphate dehydrogenase levels. primer sequences are listed in Table 1.

### 4.6. Fabrication and Characterization of PCL and ZnPCL Nanofibrous Scaffolds

Poly(ε-caprolactone) (PCL) nanofibrous scaffolds were produced as a control using the electrospinning technique. Specifically, a solution of 12% (*w*/*v*) PCL (Sigma, St. Louis, MO, USA) in a mixture of dichloromethane (DCM, Sigma) and dimethylformamide (DMF, Sigma) at a 4:6 volume ratio was electrospun. This process utilized a 21 G diameter needle, with the needle-to-drum (15 cm in diameter, rotating at 460 rpm) distance set at 15 cm and a flow rate of 1 mL/h. The PCL nanofibers were collected on aluminum foil wrapped around the drum collector. To fabricate ZnPCL scaffolds, a similar process was employed but with the inclusion of 1% (*w*/*w*) ZnO (Sigma) nanoparticles in the PCL solution. Initially, a 30% (*w*/*v*) PCL solution in DCM was prepared. Subsequently, ZnO nanoparticles were ultrasonicated in DMF for 10 min to achieve a uniform dispersion. This ZnO nanoparticle suspension was then added to the 30% (*w*/*v*) PCL solution, resulting in a final concentration of 12% (*w*/*v*) PCL. The mixture containing ZnO nanoparticles was gently stirred on a rocker overnight to ensure thorough mixing. The ZnO-infused PCL nanofibers (ZnPCL) were then fabricated using the same electrospinning parameters as those used for the PCL nanofibers

To examine the morphology using scanning electron microscopy (SEM), both PCL and ZnPCL nanofiber samples were sputter-coated with platinum under a current of 20 mA for 100 s. Their morphologies were then observed with an SEM (Auriga, Carl Zeiss, Jena, Germany). The diameters of the fibers were determined from SEM images taken at a magnification of 10,000×, using ImageJ 1.54g (National Institutes of Health, Bethesda, MD, USA). The incorporation of zinc into ZnPCL was verified through energy-dispersive spectroscopy (EDS) and thermogravimetric analysis. The tensile strength of the PCL and ZnPCL, prepared with dimensions of 10 × 60 mm^2^ (width and length), was assessed using a universal testing machine (Instron, Norwood, MA, USA) equipped with a 1000 N load cell and at a crosshead speed of 5 mm/min. Contact angles were measured using the sessile drop method at room temperature with an optical angle goniometer. The average was taken from both the right and the left contact angles of the drop.

### 4.7. In Vitro Release of Zinc Ion and Biodegradation

The sample squares measuring 10 mm × 10 mm from the ZnPCL batch were submerged in simulated body fluid (SBF) at 37 °C for a duration of 28 days. The release of zinc ions from these nanofibers was quantified using an inductively coupled plasma mass spectrometer (ICP-MS, Varian 820-MS, Varian, CA, USA). To assess the biodegradation properties of both PCL and ZnPCL, nanofiber pieces of the same dimensions were also immersed in SBF for 14 days at 37 °C. Molecular weight (Mw) analysis of the samples was conducted using gel permeation chromatography (GPC, Ultimate 3000, Thermo, Waltham, MA, USA).

### 4.8. Isolation of Mouse Neutrophils

The femur and tibia were separated from young and aged mice. The bone epiphyses of the femur and tibia were cut off and flushed using RPMI. Collected bone marrow cells were treated with RBC lysis buffer, washed with RPMI with 10% FBS, and resuspended in 1 mL of PBS. In a 15 mL tube, 3 mL of Histopaque 1119 (Sigma), 3 mL of Histopaque 1077 (Sigma), and 1 mL of cell suspension were sequentially overlaid and centrifugation was performed at 880 g for 40 min at room temperature without brake. Isolated cells were analyzed using FACS, and more than 96% were expressed Ly6g, a neutrophil marker.

### 4.9. NETosis Assay

To examine the effect of zinc on NETosis, freshly isolated neutrophils were treated with 5 uM A23187 for 4 h under various concentrations of zinc and zinc chelator. For NETosis confirmation, the DNA released due to NETosis was stained using SYTOX™ Green Nucleic Acid Stain (Invitrogen). Following staining, samples were analyzed using confocal fluorescence microscopy, image exfluore, or FACS verse.

### 4.10. Measurement of Intracellular Free Zinc Ion Level

For the measurement of free zinc levels in cells, neutrophils were incubated for 1 h under experimental conditions, stained with FluoZin™-3 (Invitrogen), and analyzed for relative zinc content using FACS Verse. Additionally, zinc levels were measured using a Zinc Assay Kit (Abcam). After incubation for 4 h, the neutrophils were lysed with EDTA-free RIPA buffer (GenDEPOT) and processed according to the manufacturer’s instructions.

### 4.11. Western Blot

After the appropriate treatment, proteins were extracted from cells using PRO-PREP™ Protein Extraction Solution (Intron Biotechnology, Seongnam, Republic of Korea) following the product protocol. SDS-PAGE with a 12% running gel was employed, and the separated proteins were electrotransferred onto a PVDF membrane. Following blocking, the membrane was incubated overnight with the Anti-Histone H3 (citrulline R2 + R8 + R17) antibody (Abcam) and anti-rabbit HRP-conjugated secondary antibody in Tris-buffered saline containing 0.1% Tween20. For β-actin, an HRP-conjugated anti-β-actin antibody (Santa Cruz Biotechnology, Dallas, TX, USA) was employed.

### 4.12. Statistical Analysis

All quantitative data were expressed as the mean ± standard deviation. Statistical analysis was performed using one-way analysis of variance (ANOVA) with Tukey post hoc test using SPSS software 26 or Student’s *t*-test. A value of *p* < 0.05 was considered statistically significant.

## 5. Conclusions

In conclusion, this study indicates the potential of ZnO nanoparticle-infused biomaterials, specifically ZnPCL, to provide zinc ions to neutrophils, reduce excessive NETosis, facilitate M2 polarization, and enhance bone healing in aged mice. These findings are particularly promising for aging individuals, offering potential new avenues for improving bone healing and recovery in the elderly population.

## Figures and Tables

**Table 1:** RT-qPCR primer sequences.

Gene Name	Forward	Reverse	ID
*Gapdh*	CCCACTCTTCCACCTTCGATG	CGAGTTGGGATAGGGCCTCT	NM_008084
*Il1b*	TGCCACCTTTTGACAGTGATG	ATGTGCTGCTGCGAGATTTG	NM_008361
*Tnf*	TGTAGCCCACGTCGTAGCAAA	TGTGGGTGAGGAGCACGTA	NM_013693
*Slc39a6*	ATTGATGCTCGGGCTTGTCT	AGCCACCAAGCCAGGCTATT	NM_028076
*Slc39a14*	CCAAGCGCCATTGAAGTATGG	TTCCAATCGCCAAGGCTATGA	NM_028076
*Arg1*	ACAAGACAGGGCTCCTTTCA	TGAGTTCCGAAGCAAGCCAA	NM_007482
*Nos2*	GGTGAAGGGACTGAGCTGTT	ACGTTCTCCGTTCTCTTGCAG	NM_010927
*Mmp9*	TCACCATGAGTCCCTGGGA	AGCGGTACAAGTATGCCTCTGC	NM_013599

## Data Availability

The datasets used and/or analyzed during the current study are available from the corresponding author upon reasonable request.

## Supplementary Materials

The following supporting information can be downloaded at: https://www.mdpi.com/article/10.3390/ijms252312851/s1.
