# Supplementary material for: Targeting Age-Related Impaired Bone Healing: ZnO Nanoparticle-Infused Composite Fibers Modulate Excessive NETosis and Prolonged Inflammation in Aging
Source: Int J Mol Sci. 2024 Nov 29;25(23):12851. doi: 10.3390/ijms252312851 (PMC11641807; doi:10.3390/ijms252312851)

Figure S1. Effect of ZnPCL on M2 polarization in an in vivo bone defect model of aged mice. The PCL and ZnPCL scaffolds were placed in 3 mm calvarial bone defects and analyzed 7 days post-operation. Ccr7 (red) and Cd163 (green) indicate specific markers, while DAPI (blue) marks nuclei.

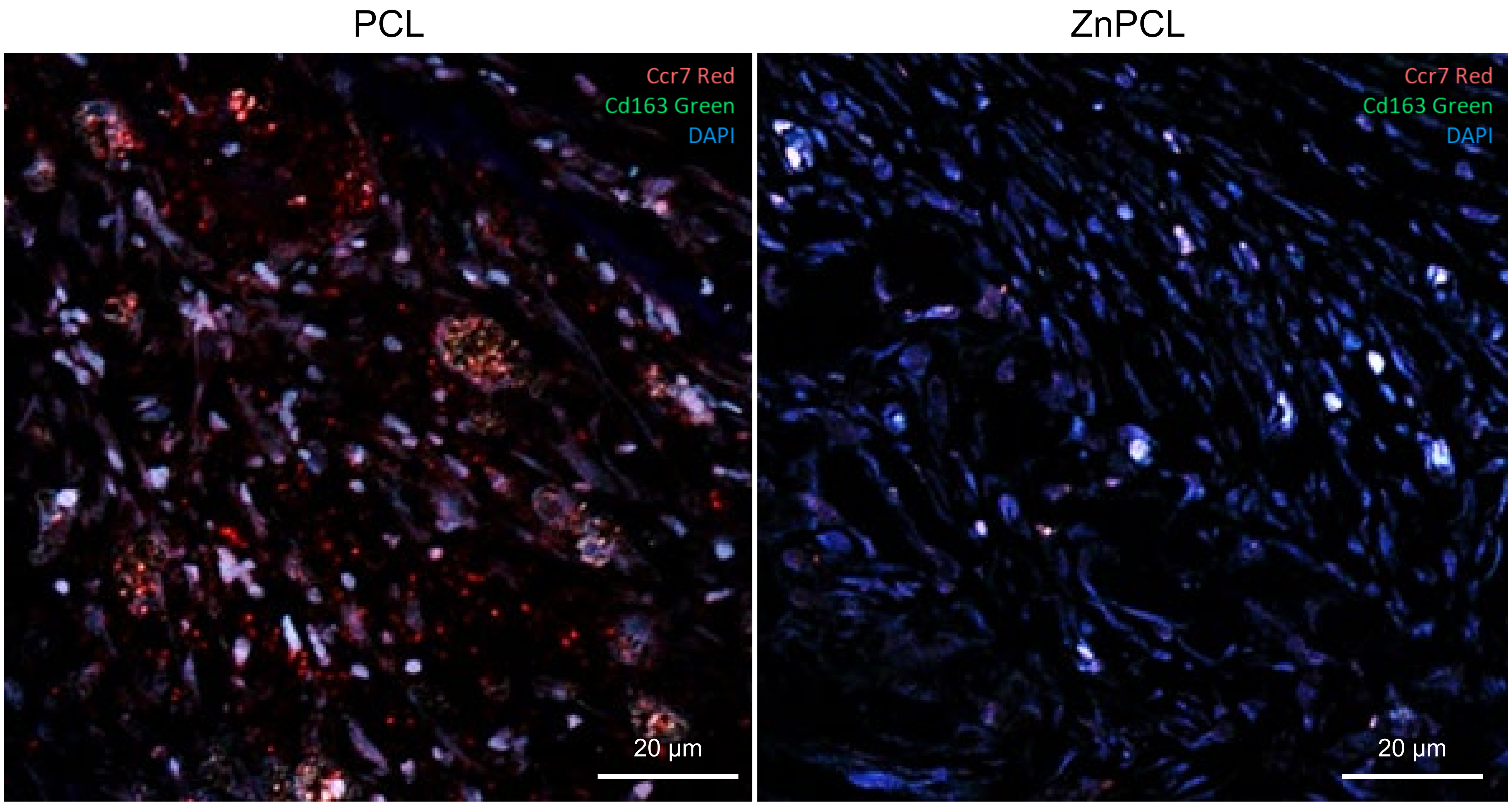

Figure S2. Effect of ZnPCL on bone healing in aged mice. Microscopic images showing bone defect healing 8 weeks after placing PCL and ZnPCL scaffolds on the defects. The images were captured at an original magnification of x2 (scale bar: 500 µm).

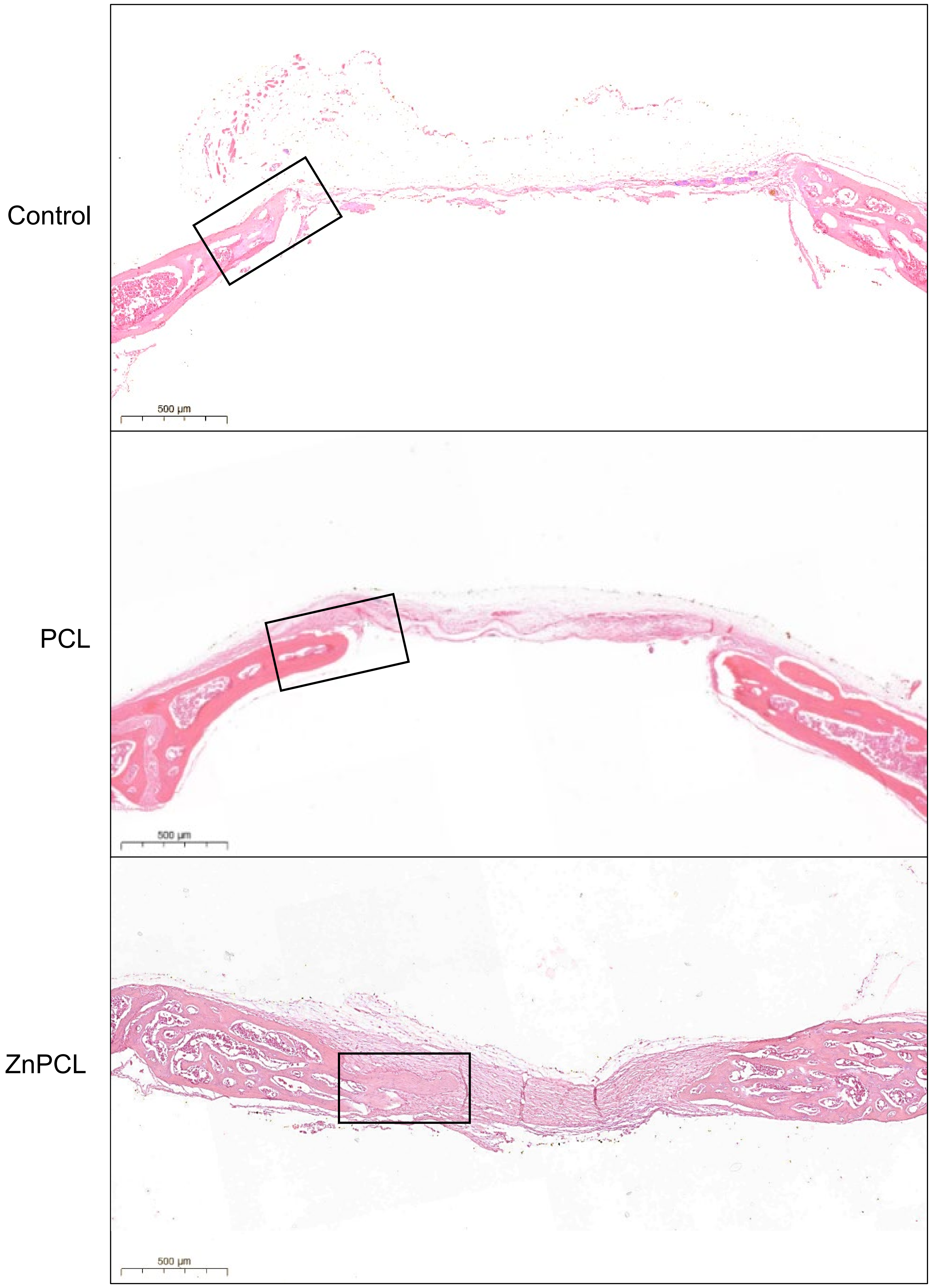

Supplement: Supplementary file 1 [file ijms-25-12851-s001.zip › ijms-3329537-supplementary.pdf]
